# Supplementary material for: Cas9-derived peptides presented by MHC Class II that elicit proliferation of CD4+ T-cells
Source: Nat Commun. 2021 Aug 24;12:5090. doi: 10.1038/s41467-021-25414-9 (PMC8384835; doi:10.1038/s41467-021-25414-9)
Supplement: Supplementary file 1 — Supplementary Information [file 41467_2021_25414_MOESM1_ESM.pdf]

Cas9 derived peptides presented by MHC Class II that elicit proliferation of $CD4^+$ T-cells

Vijaya L. Simhadri[1], Louis Hopkins[1], Joseph R. McGill[1], Brian R. Duke[2], Swati Mukherjee[2], Kate Zhang[2] and Zuben E. Sauna[1]

[1]Hemostasis Branch, Division of Plasma Protein Therapeutics, Office of Tissues and Advanced Therapies, Center for Biologics Evaluation and Research, Food and Drug Administration, Silver Spring, MD, USA. [2]Editas Medicine, Cambridge, MA 02141 USA.

Correspondence: zuben.sauna@fda.hhs.gov (Z.E. Sauna)

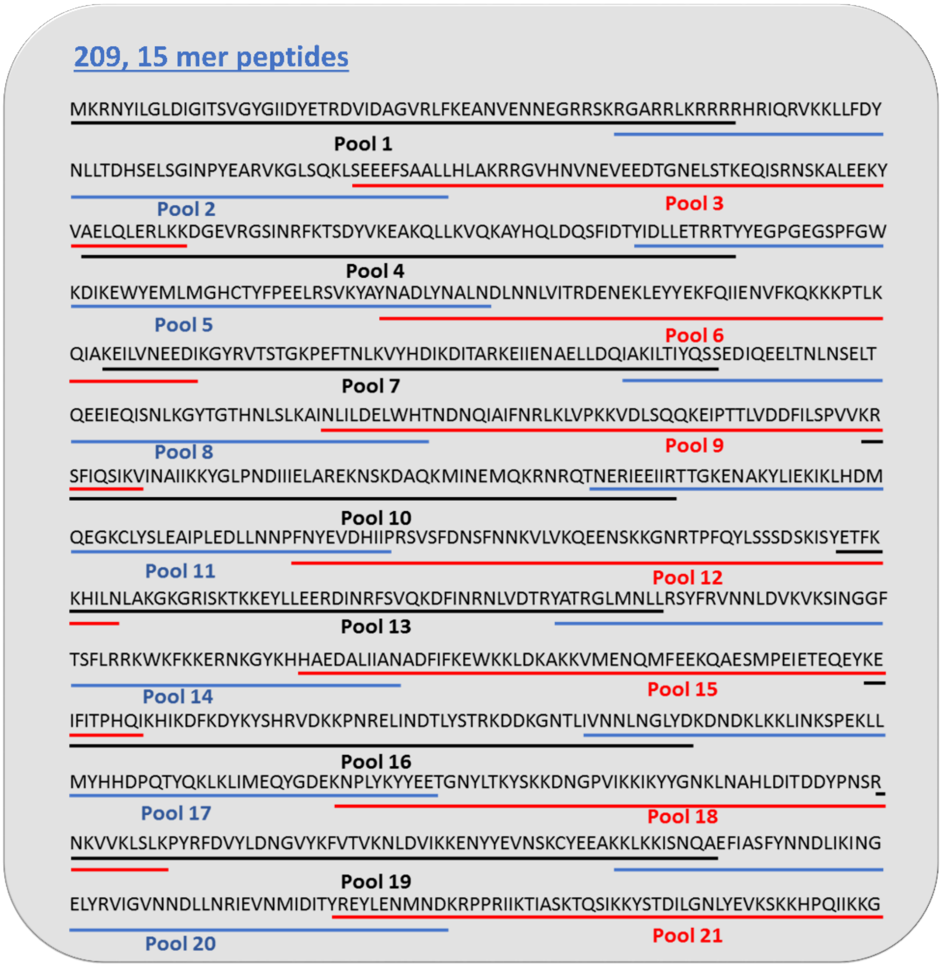

Supplementary Fig.1: SaCas9 protein sequence coverage and distribution by peptide pools

209 peptides were synthesized. Each peptide was 15 amino acids in length and peptides overlapped by 5 amino acids. Thus, the first peptide covered amino acids 1 to 15 of the SaCas9 sequence, the second amino acids 6 to 20, the third amino acids 11 to 25 and so on. Each pool contains 10 peptides. Coverage of Cas9 sequence by each peptide pool is underlined.

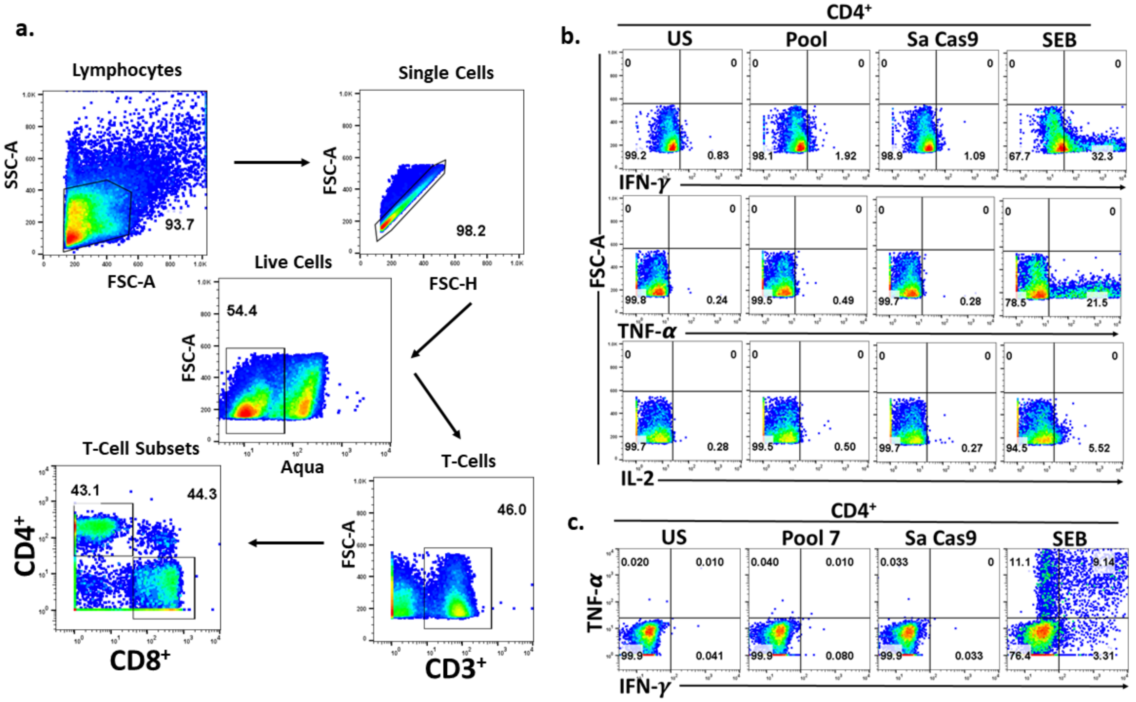

Supplementary Fig.2: Workflow and gating strategy for flow cytometry-based assay

a, Gating strategy to detect $CD4^+$ and $CD8^+$ T cells. Upregulation of cytokines were analyzed separately in the $CD4^+$ T-cells (b). **b**, Representative flow cytometry plots showing detection of antigen-induced activation measured by intracellular staining of IFN-γ, TNF-α, and IL-2, in $CD4^+$ T cells compared to unstimulated, SEB-activated, and SaCas9-activated controls. Pool 7 was selected to represent responses from PBMCs stimulated by pooled SaCas9 peptides (1 of 21). Each measured parameter was plotted against a forward side scatter area (FSC-A) y-axis. **c**, Representative figure showing IFN-γ on the x-axis and TNF-α on the Y-axis.

Donor#:	Ethnicity	Age	Sex	MHC Class I Alleles						MHC Class II Alleles	
				A		B		C		DRB1	
1	Hispanic	20	Male	26:01	29:02	44:03	50:01	06:02	16:01	03:01	07:01
2	Hispanic	38	Male	02:01	31:01	14:02	51:01	01:02	08:02	12:01	13:01
3	Hispanic	34	Male	02:02	34:02	08:01	18:01	05:01	07:01	03:01	04:03
4	Hispanic/Caucasian	39	Female	02:01	25:01	18:01	56:01	01:02	12:03	01:01	15:01
5	African/American	23	Male	02:01	68:01	07:02	15:03	02:10	07:02	13:02	15:01
6	African/American	61	Male	01:01	74:01	08:01	35:01	04:01	07:01	04:01	15:03
7	Caucasian	35	Male	01:01	02:06	15:01	37:01	01:02	06:02	08:01	11:01
8	Hispanic	33	Female	02:01	23:01	50:01	51:01	05:01	07:01	13:01	13:03
9	Caucasian	59	Male	29:02	32:01	44:03	45:01	06:02	16:01	07:01	07:01
10	Hispanic/Latino	39	Female	03:01	31:01	07:02	27:05	02:02	07:02	04:07	16:01
11	Caucasian	46	Male	02:01	29:02	44:02	44:03	05:01	16:01	04:01	07:01
12	African/American	56	Female	33:03	66:01	35:01	58:02	04:01	06:02	08:04	11:01
13	African/American	35	Male	03:01	30:02	07:02	07:02	07:02	07:02	09:01	15:01
14	African/American	31	Male	23:01	30:04	08:01	15:17	03:04	07:01	11:02	13:02
15	Hispanic	37	Male	24:02	24:02	08:01	39:06	07:01	07:02	03:01	08:02
16	Caucasian	24	Male	26:01	29:01	35:02	51:01	04:01	05:01	03:01	04:02
17	Hispanic/Latino	28	Female	03:01	24:02	08:01	35:01	04:01	07:02	01:01	11:04
18	Hispanic	38	Female	03:01	23:01	15:03	44:03	02:10	16:01	04:04	07:01
19	African/American	40	Male	24:02	36:01	44:03	52:01	04:01	07:02	04:05	12:02
20	Caucasian	44	Female	03:01	33:01	14:02	38:01	08:02	12:03	01:02	13:01
21	Caucasian	52	Male	02:01	02:01	40:01	49:01	03:04	07:01	01:01	15:01

Supplementary Table 1: Demographics (age, sex, and ethnicity) and HLA typing of donors who of PBMCs used in T cell proliferation assays.

#	Marker	Immunological Function
1	**CD3**	T-cell co-receptor, CD4+ and CD8+ T-cells
2	**CD4**	T-cell co-receptor; MCH-II restricted T-cell activation (CD4+)
3	**CD8**	T-cell co-receptor; MCH-I restricted T-cell activation (CD8+)
4	**IFN-γ**	Class II interferon cytokine, induces MCH-II expression, produced by CD4(Th1) T cells and CD8 cytotoxic T lymphocyte (CTL)
5	**TNFα**	Cytokine produced by CD4+ lymphocytes; downstream effects include promoting cellular survival and proliferation
6	**IL-2**	Cytokine typically produced by activated CD4+ and CD8+ T cells, promotes differentiation of T cells and clonal expansion
11	**FoxP3**	Member of the FOX protein family, transcription factor typically associated with the development and function of Tregs

Supplementary Table 2: List of selected markers analyzed through flow cytometry and their immunological functions. The markers that are specific to T cells (CD3, CD4, and CD8), Th1 response cytokines (IFN-γ, TNF-α, and IL-2) were used in this study.
