# Supplementary material for: Cas9-derived peptides presented by MHC Class II that elicit proliferation of CD4+ T-cells
Source: Nat Commun. 2021 Aug 24;12:5090. doi: 10.1038/s41467-021-25414-9 (PMC8384835; doi:10.1038/s41467-021-25414-9)
Supplement: Supplementary file 4 — Description of Additional Supplementary Files [file 41467_2021_25414_MOESM4_ESM.pdf]

Title: Supplementary Data 1.

Description: All Cas9 derived peptides identified in the MAPPs assay on DCs from individual donors.
