# Supplementary material for: Cas9-derived peptides presented by MHC Class II that elicit proliferation of CD4+ T-cells
Source: Nat Commun. 2021 Aug 24;12:5090. doi: 10.1038/s41467-021-25414-9 (PMC8384835; doi:10.1038/s41467-021-25414-9)
Supplement: Supplementary file 5 — Reporting Summary [file 41467_2021_25414_MOESM5_ESM.pdf]

nature portfolio

Corresponding author(s): Zuben E, Sauna

Last updated by author(s): Jul 12, 2021

Reporting Summary

Nature Portfolio wishes to improve the reproducibility of the work that we publish. This form provides structure for consistency and transparency in reporting. For further information on Nature Portfolio policies, see our Editorial Policies and the Editorial Policy Checklist.

Statistics

For all statistical analyses, confirm that the following items are present in the figure legend, table legend, main text, or Methods section.

n/a	Confirmed	
☐	☒	The exact sample size (*n*) for each experimental group/condition, given as a discrete number and unit of measurement
☐	☒	A statement on whether measurements were taken from distinct samples or whether the same sample was measured repeatedly
☐	☒	The statistical test(s) used AND whether they are one- or two-sided<br>*Only common tests should be described solely by name; describe more complex techniques in the Methods section.*
☒	☐	A description of all covariates tested
☐	☒	A description of any assumptions or corrections, such as tests of normality and adjustment for multiple comparisons
☒	☐	A full description of the statistical parameters including central tendency (e.g. means) or other basic estimates (e.g. regression coefficient) AND variation (e.g. standard deviation) or associated estimates of uncertainty (e.g. confidence intervals)
☒	☐	For null hypothesis testing, the test statistic (e.g. *F*, *t*, *r*) with confidence intervals, effect sizes, degrees of freedom and *P* value noted<br>*Give P values as exact values whenever suitable.*
☒	☐	For Bayesian analysis, information on the choice of priors and Markov chain Monte Carlo settings
☒	☐	For hierarchical and complex designs, identification of the appropriate level for tests and full reporting of outcomes
☒	☐	Estimates of effect sizes (e.g. Cohen's *d*, Pearson's *r*), indicating how they were calculated

Our web collection on statistics for biologists contains articles on many of the points above.

Software and code

Policy information about availability of computer code

Data collection	Flow cytometry data collection was performed with BD FACSDiva (v6)
Data analysis	Data Analysis was done in R v3.6.1. Base R functions were used for Fisher testing. Graphics were created using ggplot2 (v3.3.0), cowplot (v1.0.0) , reshape2 (v1.4.4) Code is available from the corresponding author with a request. Flow Cytometry Data Analysis was performed with FlowJo (v10.5.3). SampPick Software to select a representative donor cohort can be found at https://www.github.com/fda/SampPick.

For manuscripts utilizing custom algorithms or software that are central to the research but not yet described in published literature, software must be made available to editors and reviewers. We strongly encourage code deposition in a community repository (e.g. GitHub). See the Nature Portfolio guidelines for submitting code & software for further information.

Data

Policy information about availability of data

All manuscripts must include a data availability statement. This statement should provide the following information, where applicable:

- Accession codes, unique identifiers, or web links for publicly available datasets
- A description of any restrictions on data availability
- For clinical datasets or third party data, please ensure that the statement adheres to our policy

The data generated in this study have been deposited in the Harvard Dataverse Repository and can be accessed at https://doi.org/10.7910/DVN/O4PUO1. The following 5 files have been provided in the repository. File 1: Background frequencies of North American HLA-DRB1 alleles; File 2: Flow cytometry, MFI for cytokines; File 3: Flow cytometry, cell counts for each marker; File 4: HLA typing of donors used as a source of cells for follow cytometry; File 5: Mass spectrometric data for the MHC Associated Peptide Proteomics assay. The datasets used to generate each of the figures are: Figure 1a (Files 1, 4, 5); Figures 1b,c & Figures 2a,b (Files 2, 3); Figure 3 (Files 1,2,3,4); Figure 4a,b (File 5); Figure 4c (Files 2,3,4,5).

Field-specific reporting

Please select the one below that is the best fit for your research. If you are not sure, read the appropriate sections before making your selection.

☒ Life sciences ☐ Behavioural & social sciences ☐ Ecological, evolutionary & environmental sciences

For a reference copy of the document with all sections, see nature.com/documents/nr-reporting-summary-flat.pdf

Life sciences study design

All studies must disclose on these points even when the disclosure is negative.

Sample size	Donors for PBMCs (21) were collated from a larger pool of 50 MHC-typed donors using SampPick, an algorithm we developed recently (ref 16 in manuscript). SampPick optimizes cohort selection to closely match the frequency distribution of MHC variants in the target population; in this study that was the North American population. No sample size calculations were necessary for this study. The only requirement was that donor derived cells reflected the MHC distribution observed in the North American population.
Data exclusions	No data was excluded.
Replication	As it was not possible to re-assemble the same cohort for repeating the experiment. The size of the cohort (21) represent replicates for each treatment (stimulation with individual Cas9 peptides or proteins). Similarly for the MAPPs assay Cas9 derived peptides were identified from antigen presenting cells obtained from 18 individual donors. The 21 donors derived cells used in the flow cytometry experiments and 18 donor derived cells use in the MAPPs assay are not expected to respond in the same way due to the variation in their MHC repertoire. This has been taken into account while analyzing the data.
Randomization	Randomization was not relevant to this study. This is not a clinical study. Moreover this study is does not have two or more arms. All donor samples were subjected to Cas9 protein or peptides.
Blinding	There was no blinding of subjects in this study. This is an experimental study with a single arm and all donor derived PBMCs were treated with the Cas9 peptides or proteins. Knowing which protein or peptide each donor was treated with as well as the HLA type of each donor was required for the analysis. The same individual carried out the experiments and analyses hence blinding was not feasible.

Reporting for specific materials, systems and methods

We require information from authors about some types of materials, experimental systems and methods used in many studies. Here, indicate whether each material, system or method listed is relevant to your study. If you are not sure if a list item applies to your research, read the appropriate section before selecting a response.

Materials & experimental systems

n/a	Involved in the study	
☐	☒	Antibodies
☒	☐	Eukaryotic cell lines
☒	☐	Palaeontology and archaeology
☒	☐	Animals and other organisms
☒	☐	Human research participants
☒	☐	Clinical data
☒	☐	Dual use research of concern

Methods

n/a	Involved in the study	
☒	☐	ChIP-seq
☐	☒	Flow cytometry
☒	☐	MRI-based neuroimaging

Antibodies

Antibodies used	All antibodies were purchased from Becton, Dickinson & Co.(BD). Catalog numbers are given in parentheses: Hu CD3 APC-Cy7 SK7 (557832); Hu CD4 BV711 SK3 (563028); Hu CD8 BV786 RPA-T8 (563823); Hu IFN-Gma BV421 B27 (562988), Hu IL-2 BV650 5344.111 (563467); Hu TNF PE-CF594 Mab11 (562784); CD28/CD29d co-stimulatory reagent (347690).
Validation	Antibodies were commercially available through BD, and previously published (according to manufacturers website). Citations for use of BD manufactured antibodies are below:<br>Nature Med. February 2019 Vol.25 (242-248)<br>Nature Med. February 2019 Vol.25 (249-254)

Flow Cytometry

Plots

Confirm that:

- [x] The axis labels state the marker and fluorochrome used (e.g. CD4-FITC).
- [x] The axis scales are clearly visible. Include numbers along axes only for bottom left plot of group (a 'group' is an analysis of identical markers).
- [x] All plots are contour plots with outliers or pseudocolor plots.
- [x] A numerical value for number of cells or percentage (with statistics) is provided.

Methodology

Sample preparation	Peripheral blood mononuclear cells (PBMCs) were collected, prepared and cryopreserved by Cellular Technology Limited (CTL). According to their sources, PBMCs were isolated and collected from leukocytes through leukapharesis and frozen in CTL-Cryo ABC serum-free freezing medium. Samples are collected in full compliance with HIPAA guidlines.
Instrument	LSRFortessa X-20 flow cytometer (BD Biosciences, San Jose, CA)
Software	FlowJo (v10.5.3) (Tree Star)
Cell population abundance	No sorting was performed for this study.
Gating strategy	Lymphocytes were gated based on FSC verse SSC profile, and doublets were excluded using FSC-A plotted against FSC-Height (FSC-H). Once single cells were selected, the samples were further gated for alive cells by selecting populations negative for LIVE/DEAD Fixable Aqua. To identify T cell populations, CD3+, CD4+ cells or CD3+, CD8+ cells were gated for analysis of various intracellular markers (CD137, CD154, IFN-γ, TNF-α, and IL-2). Unstimulated cells were used as controls. Gates were set according to the unstimulated control in order to establish positive parameters and background responses.

- [x] Tick this box to confirm that a figure exemplifying the gating strategy is provided in the Supplementary Information.
